# Supplementary material for: A targeted genetic modifier screen in Drosophila uncovers vulnerabilities in a genetically complex model of colon cancer
Source: G3 (Bethesda). 2023 Mar 6;13(5):jkad053. doi: 10.1093/g3journal/jkad053 (PMC10151408; doi:10.1093/g3journal/jkad053)
Supplement: jkad053_Supplementary_Data [file jkad053_supplementary_data.zip › Supplemental_Figure_Legends_G3-2023-404065.docx]

**Figure S1. Genetic crosses for the screens.** (A-D) Example crosses for KRAS TP53 PTEN APC (A,B) and KRAS (C,D) screens for kinases on the second chromosome (see methods for additional details). Experimental Pupae (EP) were identified by the absence of the Tb marker. Experimental Adults (EA) were scored by identifying empty experimental pupal cases, indicating an adult fly has emerged. (E,F). Images of vials from the screens that show example EP’s, CP’s and EA’s.

**Figure S2. Additional controls for validating hits from the screens.**  (A) Baseline controls for lethality of mutant alleles identified as hits from both screens in a GFP-only (*byn>UAS-GFP*), otherwise wildtype background. (B,C) Rescue of KRAS TP53 PTEN APC (B) and KRAS (C)-induced lethality using a different set of alleles for the hits. Mutant alleles: *aPKC^MI10848^, par-1^k05603^, cdk2^3^, hppy^MI03637^, dsor^G42^, akt^MI14526^***.** Error bars represent Standard Error of the Mean (SEM). * p≤0.05, ** p≤0.01, **** p≤0.0001 (Multiple unpaired t-tests with Holm-Sidak Correction, PRISM Software). (D) Quantification of the imaginal ring area of original hits (Figure 2A,B) in a GFP-only (*byn>UAS-GFP*) control background (one-way ANOVA, PRISM Software). (E) Representative images used for the quantification of the imaginal ring region of the hindgut for the analyses presented in Figure 2E,F. Hindgut epithelium in green, quantified area outlined by yellow dashed lines.

**Figure S3. Additional controls for the knock-down analyses.** (A) Representative images used for the quantification of the imaginal ring region of the hindgut for the analyses presented in Figure 3E-H. Hindgut epithelium in green, quantified area outlined by yellow dashed lines. (B,C) Quantification of KRAS TP53 PTEN APC induced lethality  (B) and imaginal ring area (C) upon knock down of *akt* and *dsor* using an independent set of RNAi lines and *luciferase* RNAi as a negative control. Error bars represent Standard Error of the Mean (SEM). **** p≤0.0001 (one-way ANOVA, PRISM Software).
