# Supplementary figures and images for: A targeted genetic modifier screen in Drosophila uncovers vulnerabilities in a genetically complex model of colon cancer
Source: G3 (Bethesda). 2023 Mar 6;13(5):jkad053. doi: 10.1093/g3journal/jkad053 (PMC10151408; doi:10.1093/g3journal/jkad053)

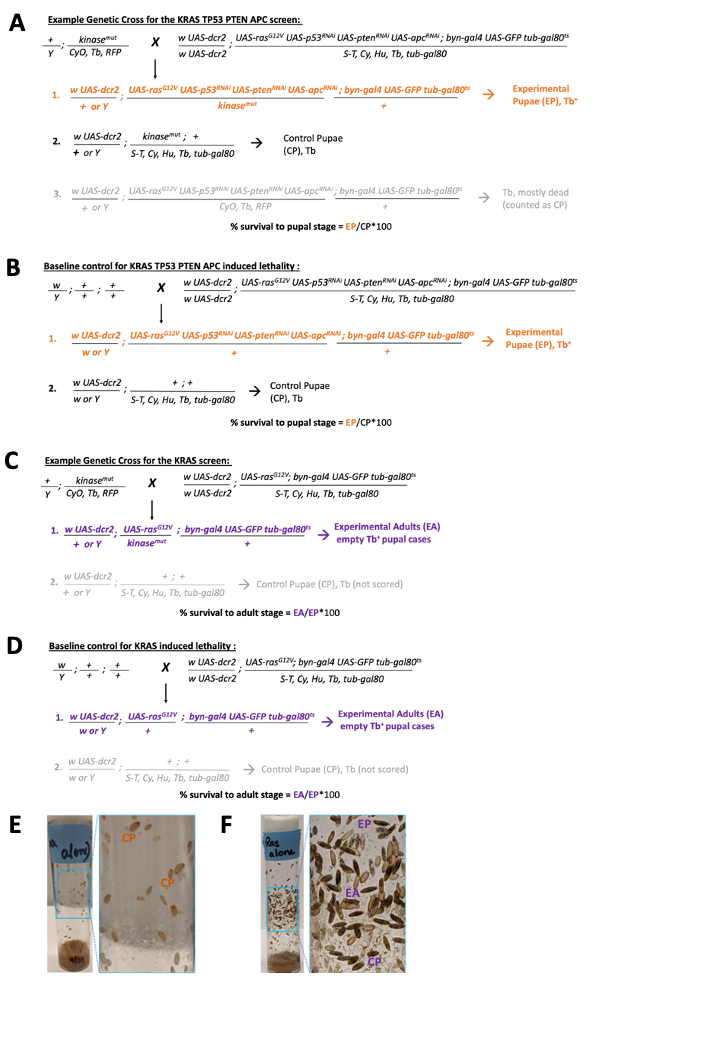

Supplement: jkad053_Supplementary_Data [file jkad053_supplementary_data.zip › Figure_S1_G3-2023-404065.tif]

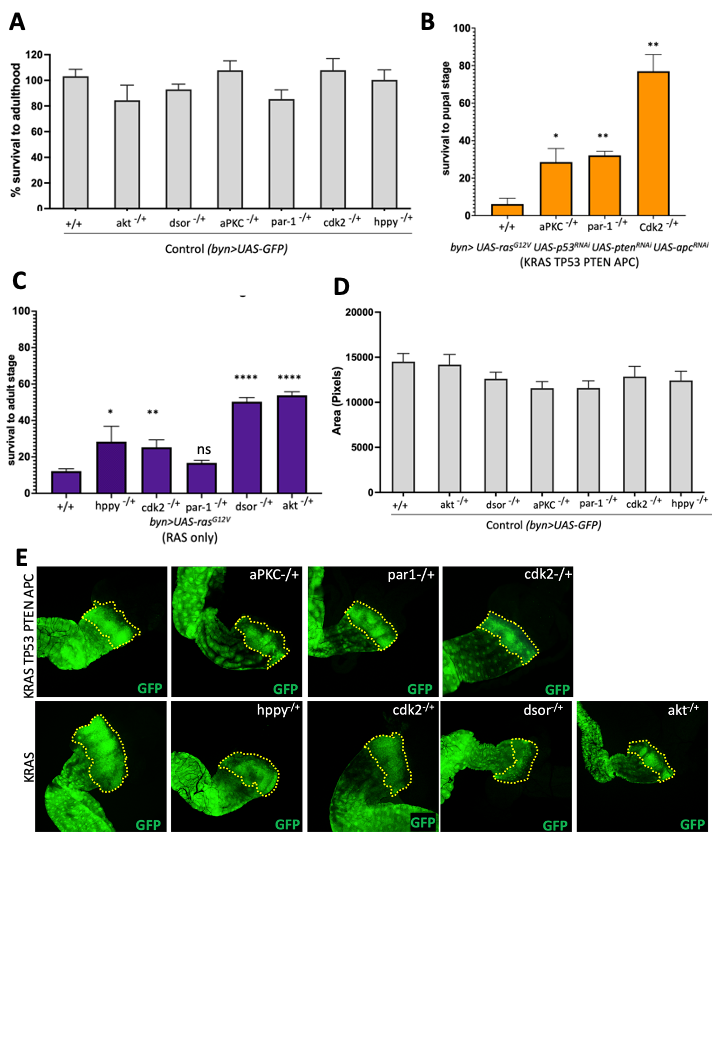

Supplement: jkad053_Supplementary_Data [file jkad053_supplementary_data.zip › Figure_S2_G3-2023-404065.tif]

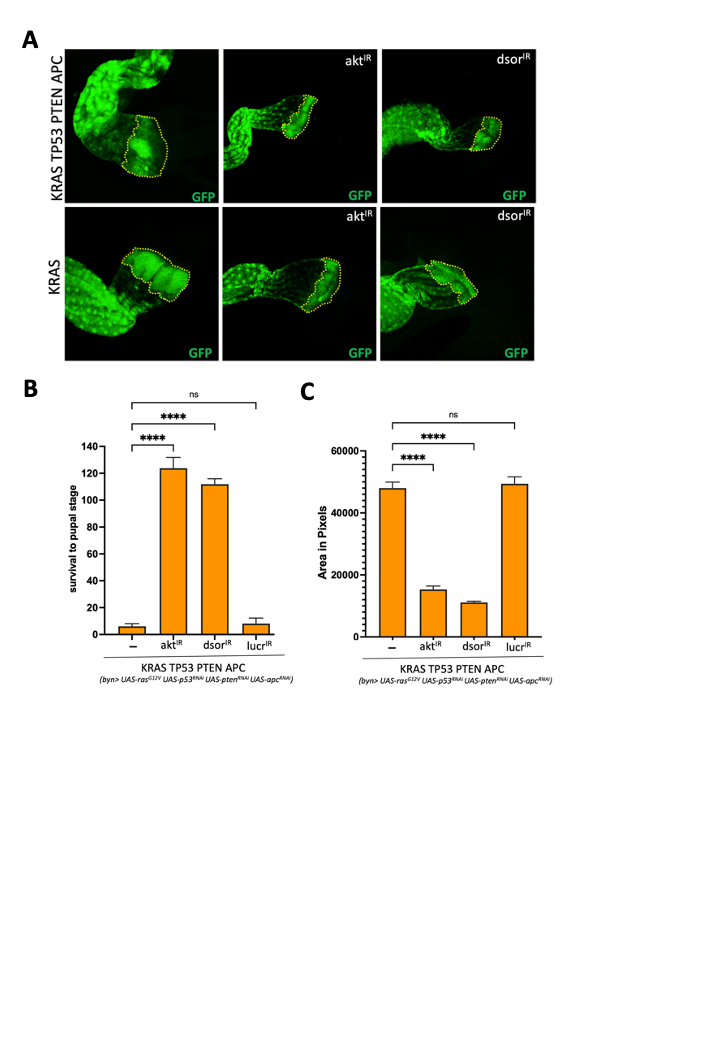

Supplement: jkad053_Supplementary_Data [file jkad053_supplementary_data.zip › Figure_S3_G3-2023-404065.tif]
